# TDP-43 knockdown impairs neurite outgrowth dependent on its target histone deacetylase 6

**DOI:** 10.1186/1750-1326-6-64

**Published:** 2011-08-30

**Authors:** Fabienne C Fiesel, Christine Schurr, Stephanie S Weber, Philipp J Kahle

**Affiliations:** 1 Laboratory of Functional Neurogenetics, Department of Neurodegeneration, Hertie Institute for Clinical Brain Research, Otfried-Mueller-Str. 27, Tuebingen, 72076, Germany; 2 German Center for Neurodegenerative Diseases, University of Tuebingen, Otfried-Mueller-Str. 27, Tuebingen, 72076, Germany

**Keywords:** TDP-43, RNAi, HDAC6, neurite outgrowth, SH-SY5Y neuroblastoma, frontotemporal dementia, amyotrophic lateral sclerosis

## Abstract

**Background:**

Trans-activation response element (TAR) DNA binding protein of 43kDa (TDP-43) is causally related to the neurodegenerative diseases frontotemporal dementia and amyotrophic lateral sclerosis being the hallmark protein in the disease-characteristic neuropathological lesions and via genetic linkage. Histone deacetylase 6 (HDAC6) is an established target of the RNA-binding protein TDP-43. HDAC6 is an unusual cytosolic deacetylase enzyme, central for a variety of pivotal cellular functions including aggregating protein turnover, microtubular dynamics and filopodia formation. All these functions are important in the context of neurodegenerative proteinopathies involving TDP-43. We have previously shown in a human embryonic kidney cell line that TDP-43 knockdown significantly impairs the removal of a toxic, aggregating polyQ ataxin-3 fusion protein in an HDAC6-dependent manner. Here we investigated the influence of TDP-43 and its target HDAC6 on neurite outgrowth.

**Results:**

Human neuroblastoma SH-SY5Y cells with stably silenced TDP-43 showed a significant reduction of neurite outgrowth induced by retinoic acid and brain-derived neurotrophic factor. Re-transfection with TDP-43 as well as HDAC6 rescued retinoic acid-induced neurite outgrowth. In addition, we show that silencing of HDAC6 alone is sufficient to reduce neurite outgrowth of *in vitro *differentiated SH-SY5Y cells.

**Conclusions:**

TDP-43 deficiency leads to impairment of neurite growth in an HDAC6-dependent manner, thereby contributing to neurodegenerative events in TDP-43 diseases.

## Background

Trans-activation response element (TAR) DNA binding protein of 43kDa (TDP-43) is the neuropathological hallmark protein of a new class of neurodegenerative dementias and movement disorders comprising certain types of frontotemporal lobar atrophy (FTLD-TDP) and amyotrophic lateral sclerosis (ALS) [1]. There is also established genetic linkage to these diseases [2]. Thus, TDP-43 is causally implicated in the pathogenesis of these neurodegenerative diseases, but the mechanism(s) are largely unknown.

TDP-43 was originally identified as a protein binding to TAR DNA sequences within human immunodeficiency virus type 1 and acting as a strong transcriptional repressor [3]. In addition to potential transcriptional regulation, TDP-43 affects a number of identified RNAs [4]. TDP-43 regulates splicing of the pre-mRNAs for cystic fibrosis transmembrane conductance regulator [5], apolipoprotein A2 [6], survival of motor neuron protein [7], and splicing component of 35kDa [8], as well as the processing of miRNAs [9]. TDP-43 has been reported to regulate low molecular weight neurofilament mRNA stability [10]. Recent microarray screens identified histone deacetylase 6 (HDAC6) as an altered transcript in TDP-43 silenced cells [11] and in conditional knockout mice [12]. Moreover, *HDAC6 *was consistently identified by systematic sequencing of RNA isolated by crosslinking immunoprecipitation using TDP-43 antibodies [13,14]. TDP-43 binds to *HDAC6 *mRNA and regulates its expression [11,15].

HDAC6 is an unusual, cytosolic deacetylase with manifold cellular functions. For example, HDAC6 is centrally involved in misfolded protein and organelle degradation processes [16]. HDAC6 regulates protein chaperone activities by acting as a deacetylase of heat shock protein of 90kDa (HSP90). In conjunction with another gene product (valosin-containing protein) associated with a form of FTD (inclusion body myopathy with Paget disease of bone and frontotemporal dementia) and ALS [17,18], HDAC6 decides over proteasomal versus autophagic breakdown fates [19]. Indeed, we have previously shown that HDAC6 down-regulation after TDP-43 silencing impairs the turnover of toxic aggregating proteins [11]. Moreover, we demonstrated an accumulation of one of the major HDAC6 substrates, acetyl-tubulin [11]. As HDAC6 also deacetylates cortactin, cytoskeletal and motility defects [20] may occur in TDP-43 deficient cells. With regard to the neurodegenerative disease aspect of TDP-43, we addressed the question if TDP-43 down-regulation might impair neurite outgrowth in a manner involving HDAC6.

## Results

### Reduction of neurite outgrowth by TDP-43 knockdown

**Figure 1:**
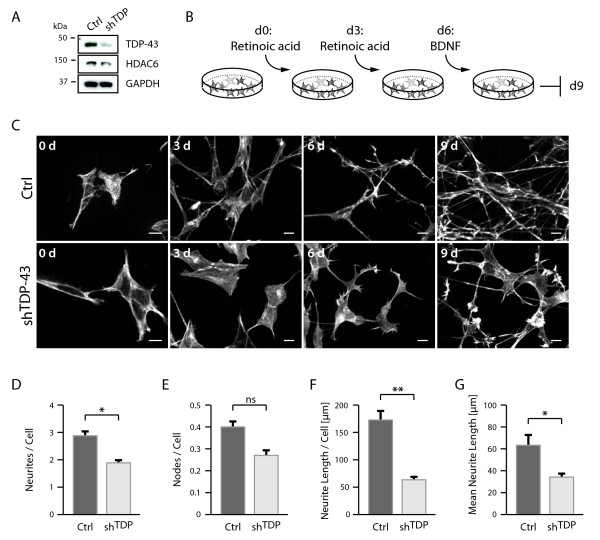
**Reduced neurite outgrowth in sh^TDP ^cells**. A, Parental SH-SY5Y cells (Ctrl) or cells stably transduced with shRNA against TDP-43 (sh^TDP^) were lysed, electrophoresed and Western blots sequentially probed with antibodies against TDP-43 (top panel) and HDAC6 (middle panel). Anti-GAPDH probing (bottom panel) confirmed equal loading. B, Schematic protocol for neurite outgrowth. Cells were primed with RA for 3d, after 3d RA-containing medium was changed. After another 3d medium was changed to serum-free supplemented with 50 ng/ml BDNF followed by further incubation. C, After each indicated interval, some cover slips were taken for fixation and staining with Alexa568-phalloidin. Size bars correspond to 10 μm. Quantifications were performed for the parameters D, number of neurites per cell, E, number of neurite branches per cell, F, total neurite length per cell and G, mean neurite length. All parameters were reduced in sh^TDP ^cells (light bars) compared to control cells (dark bars), either showing a trend (ns) or significantly (*p < 0.05, **p < 0.005).

Western blot analysis confirmed [11] the reduction of TDP-43 and HDAC6 protein in sh^TDP ^cells stably expressing TDP-43 directed shRNA compared to control parental SH-SY5Y cells (Figure 1A). Neuronal differentiation was induced by treatment with retinoic acid (RA) and brain-derived neurotrophic factor (BDNF) (Figure 1B). After 3d RA treatment, control cells grew appreciable neurites, which formed robust neuritic networks after the RA-BDNF differentiation protocol (Figure 1C). In contrast, sh^TDP ^cells barely induced neurites after 3d RA treatment, and formed much reduced neuritic networks during the RA-BDNF treatment (Figure 1C). Instead, the phalloidin stainings of actin filaments often showed abnormal growth cone structures and stress fibers in differentiated sh^TDP ^cells (Figure 1C). Compared to control cells, sh^TDP ^cells had significantly less neurites per cell (Figure 1D), less neurite branches (Figure 1E), and significantly shorter neurites (Figure 1F and 1G).

### Neurite outgrowth impairment depends on TDP-43 and HDAC6

Re-transfection experiments were performed to assess whether neurite growth impairments in sh^TDP ^cells depended directly on TDP-43 and HDAC6. Transfection of TDP-43 did not lead to high overexpression levels of TDP-43 (see additional file 1A for Western blots and additional file 1B for densitometric quantification) probably reflecting the previously reported self-regulation of TDP-43 [21]. Thus, TDP-43 re-transfection may not full restore normal functional TDP-43 protein levels. Nevertheless, TDP-43 transfection was sufficient to completely restore HDAC6 levels in sh^TDP ^cells (see additional file 1A for Western blots and additional file 1C for densitometric quantification).

**Figure 2:**
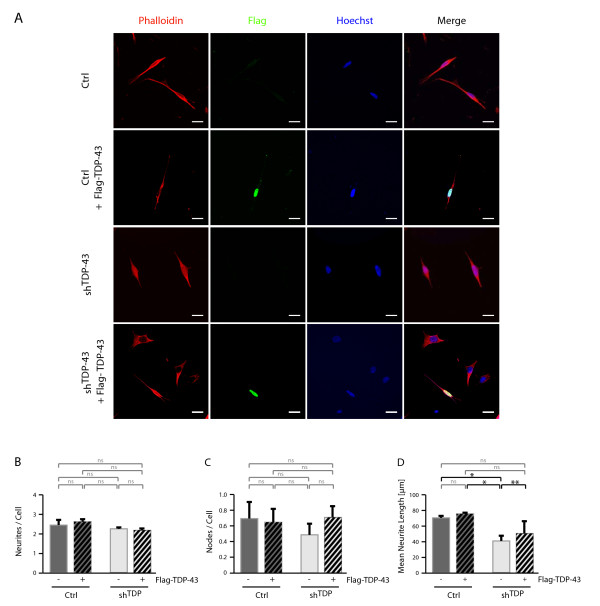
**Rescue of neurite outgrowth in sh^TDP ^cells by TDP-43 re-transfection**. Parental SH-SY5Y cells (Ctrl) or cells stably transduced with shRNA against TDP-43 (sh^TDP^) were exposed to RA. After 1d, cells were transfected for 4h with Flag-TDP-43 or control vector. After another 3d RA treatment, cells were fixed and labeled with Alexa568-phalloidin (red) and anti-Flag (green). Nuclei were counterstained with Hoechst (blue). A, shown are representative images. Scale bar corresponds to 20 μm. Quantifications of re-transfected, Flag-positive cells were performed for the parameters B, number of neurites per cell, C, number of neurite branches and D, mean neurite length. Significance levels are indicated as in Figure 1.

**Figure 3:**
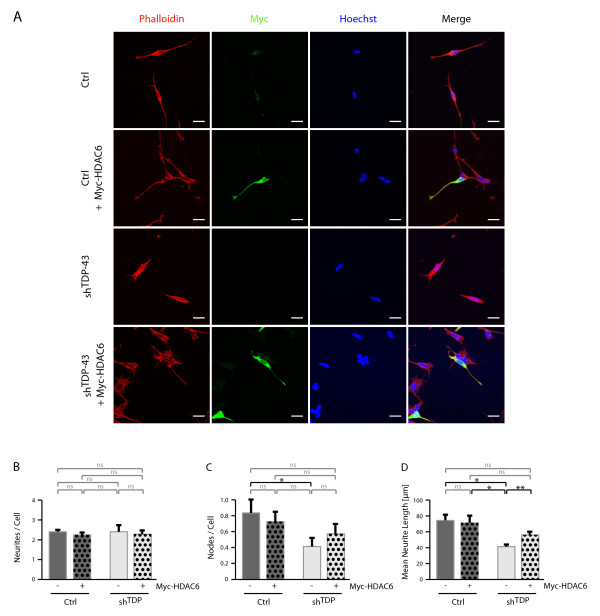
**Rescue of neurite outgrowth in sh^TDP ^cells by HDAC6 transfection**. Parental SH-SY5Y cells (Ctrl) or cells stably transduced with shRNA against TDP-43 (sh^TDP^) were exposed to RA. After 1d, cells were transfected for 4h with Myc-HDAC6 or control vector. After another 3d RA treatment, cells were fixed and labeled with Alexa568-phalloidin (red) and anti-Myc (green). Nuclei were counterstained with Hoechst (blue). A, shown are representative images. Scale bar corresponds to 20 μm. Quantifications of transfected, Myc-positive cells were performed for the parameters B, number of neurites per cell, C, number of neurite branches and D, mean neurite length. Significance levels are indicated as in Figure 1.

To accommodate the shorter time frames for transient re-transfections, cells were differentiated only with RA for 4d. Under these conditions, we observed no significant difference in the number of neurites per cell (Figure 2A and 3A and for quantification 2B and 3B). However, there was a trend of reduced number of neurite branches (Figure 2C and 3C) that appeared to be rescued by transfection of TDP-43 (Figure 2C) and HDAC6 (Figure 3C), but these effects did not reach statistical significance. Consistently, the reduction in neurite length could be rescued by TDP-43 re-transfection (Figure 2D), demonstrating that neurite outgrowth impairment in sh^TDP ^cells is directly related to TDP-43 depletion. Importantly, HDAC6 re-transfection significantly rescued neurite length (Figure 3D), indicating that the HDAC6 down-regulation in sh^TDP ^cells is involved in neurite outgrowth impairment. As the neurite outgrowth rescue was found to be only partial, it is possible that TDP-43 deficiency affects additional pathway(s) beyond HDAC6, which remain to be identified.

### Reduction of neurite outgrowth by HDAC6 knockdown

**Figure 4:**
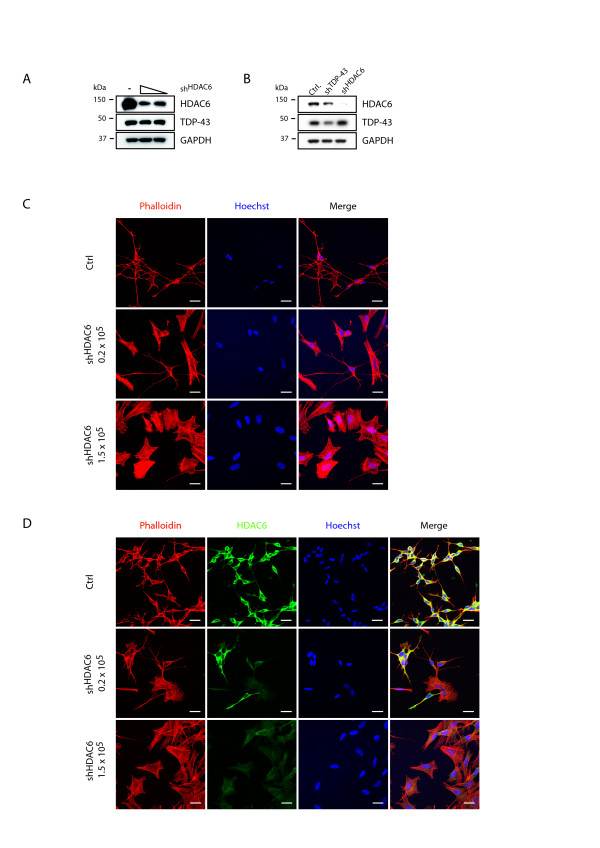
**sh^HDAC6 ^strongly reduces neurite outgrowth in SH-SY5Y cells**. Parental SH-SY5Y cells were left untreated (Ctrl) or treated with 0.2 or 1.5 × 10^5 ^TU of lentiviral particles encoding for shRNA against HDAC6 (sh^HDAC6^). TDP-43 silenced sh^TDP ^cells were included for comparison (B). A and B, Western blots show the protein levels of HDAC6 (upper panel) and TDP-43 (middle panel). GAPDH was used as a loading control. C, shown are representative images of cells that were treated with the neurite outgrowth protocol indicated in Figure 1B and stained with Alexa568-phalloidin (red). Nuclei were counterstained with Hoechst (blue). D, cells were exposed to RA for 4d, fixed and labeled with Alexa568-phalloidin (red) and anti-HDAC6 (green). Nuclei were counterstained with Hoechst (blue). Scale bars correspond to 20 μm.

To confirm that depletion of HDAC6 significantly contributes to defective neurite outgrowth of SH-SY5Y cells, we have generated stably silenced HDAC6 cells (sh^HDAC6^) by treating parental cells with different amounts of lentiviral shRNA against HDAC6. This resulted in dose-dependent decrease of HDAC6 protein (Figure 4A). The reduction of HDAC6 by direct silencing was much stronger than by TDP-43 silencing (Figure 4B). In contrast to parental controls (Figure 4C, upper panel), cells treated with high amounts of HDAC6 shRNA vector showed altered cellular morphology that was accompanied by a complete loss of neurite outgrowth upon *in vitro *differentiation with RA and BDNF (Figure 4C, lower panel). Interestingly, cells treated with less virus showed an intermediate phenotype as the cellular pool contained normal shaped SH-SY5Y cells with intact neurite outgrowth as well as cells with altered morphology without neurites (Figure 4C, middle panel). Differentiation with only RA allowed for co-staining with anti-HDAC6 antibody and showed that cellular morphology and concomitant neurite outgrowth is proportional to the amount of HDAC6 protein on the single cell level (Figure 4D). Thus, efficient silencing of HDAC6 in SH-SY5Y cells leads to an abnormal cellular phenotype and loss of neurite outgrowth.

**Figure 5:**
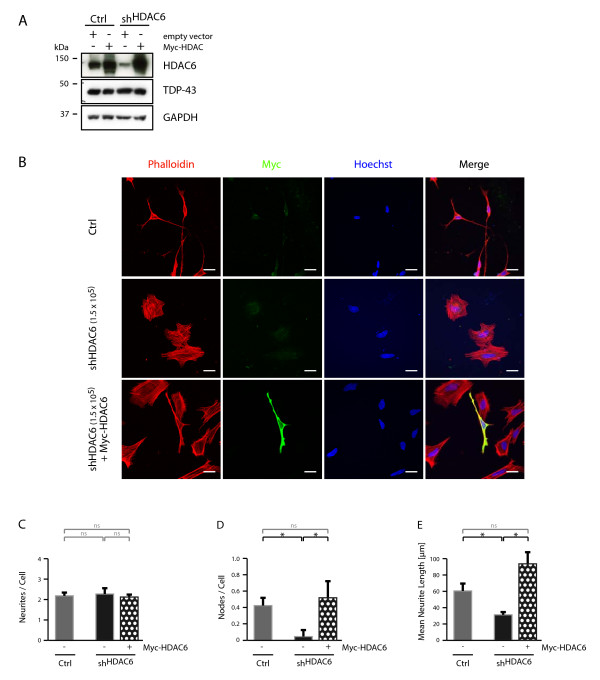
**Rescue of neurite outgrowth in sh^HDAC6 ^cells by HDAC6 transfection**. Parental SH-SY5Y cells (Ctrl) or stably transduced cells with shRNA against HDAC6 (sh^HDAC6^, 1.5 × 10^5 ^TU) were transiently transfected with Myc-HDAC6 or control vector. A, Cells were lysed, electrophoresed and Western blots sequentially probed with antibodies against HDAC6 (top panel) and TDP-43 (middle panel). Anti-GAPDH probing (bottom panel) confirmed equal loading. B, cells were exposed to RA. After 1d, cells were transfected for 4h with Myc-HDAC6 or control vector and treated for another 3d with RA. B-E, Cells were fixed and labeled with Alexa568-phalloidin (red) and anti-Myc (green). Nuclei were counterstained with Hoechst (blue). B, shown are representative images. Scale bar corresponds to 20 μm. Quantifications of transfected, Myc-positive cells were performed for the parameters C, number of neurites per cell, D, number of neurite branches and E, mean neurite length. Significance levels are indicated as in Figure 1.

In order to demonstrate that the observed defective neurite growth is dependent on HDAC6 and not an artifact of the viral shRNA transduction, we have re-introduced HDAC6 by transient transfection into sh^HDAC6 ^cells (Figure 5A). Cells were microscopically analyzed (Figure 5B) after treatment with RA for four days and neurite number, branching and length (Figure 5C, D and 5E) were quantified. Upon silencing of HDAC6, we could not observe any difference in the number of neurites (Figure 5C). However, in sh^HDAC6 ^cells branching and length of these protrusions was significantly decreased compared to control cells (Figure 5D and 5E). Overall, the phenotype of sh^HDAC6 ^cells resembled cells that were silenced for TDP-43, albeit the extent of the alterations were even stronger, which might be explained by the stronger HDAC6 downregulation by direct silencing. Importantly, HDAC6 re-expression was sufficient to reverse these drastic phenotypes completely (Figure 5), suggesting that indeed the HDAC6 protein amount in single cells is decisive for neurite outgrowth of *in vitro *differentiated SH-SY5Y cells.

## Discussion

The present study confirms the neurite outgrowth impairment in TDP-43 stably silenced human neuroblastoma SH-SY5Y cells, as was previously reported for transiently silenced mouse neuroblastoma Neuro-2a cells [22] and NSC-34 motor neuron cells [23]. Moreover, TDP-43 deficient *Drosophila melanogaster *have a similar impairment in neuritic complexity of motoneurons and neuromuscular junctions [24,25]. Thus, TDP-43 deficiency, as it may occur in human disease by cytosolic sequestration of this nuclear protein [26], causes a neurite defect (in addition to deregulated aggregating toxic protein turnover [11]) that may contribute to motorneuron disease in ALS, and if occurring in the frontal and temporal cortex also to FTLD-TDP.

Although the overall phenotype of HDAC6 knockout mice is very mild despite abnormal acetylation levels of tubulin and HSP90, at least in testis and spleen [27], a more recent report shows neurodegeneration in HDAC6 depleted mice and flies, which is accompanied by the accumulation of ubiquitinated proteins due to impaired autophagy [28] generally reminiscent of human disease. Conversely, HDAC6 promotes neuroprotection against aggregating protein toxicity [29]. Pharmacological inhibition of HDAC6 slows down axonal growth due to microtubular impairments [30]. Iguchi et al. [22] correlated the neurite outgrowth deficiencies in TDP-43 silenced mouse neuroblastoma cells to reduced activity of Rho GTPases. Thus, it is noteworthy that *Hdac6*^-/- ^mouse embryonic fibroblasts showed reduced activity of the Rho-like GTPase Rac1, which was correlated to the HSP90 deacetylase activity of HDAC6 [31]. Finally, HDAC6 was very recently shown to regulate dendrite morphogenesis in postmitotic neurons by acting on the anaphase-promoting complex and CDC20 at centrosomes [32]. Our new finding that TDP-43 mediates neurite outgrowth through HDAC6 provides a novel avenue to the understanding of neuronal signaling pathways contributing to neurodegenerative diseases.

## Conclusions

TDP-43 deficiency causes impaired neurite outgrowth. TDP-43 silencing downregulates HDAC6 levels, and transfection of HDAC6 into sh^TDP ^cells restores neurite outgrowth. Silencing HDAC6 directly causes severe cytoskeletal rearrangements and loss of neurite outgrowth in human neuroblastoma SH-SY5Y cells. Thus, TDP-43 and HDAC6 are in a linear cascade mediating neurite outgrowth. Disturbing this pathway in human TDP proteinopathies may contribute to neurodegeneration.

## Methods

### Cell Culture

Human neuroblastoma SH-SY5Y cells (ATCC) were grown in Dulbecco's modifies eagle medium: F12 (Biochrom AG) supplemented with 10% fetal bovine serum (PAA Laboratories) under humidified conditions at 37°C and 5%CO_2_. Stably silenced sh^TDP ^SH-SY5Y cells were described previously [11]. Stably silenced sh^HDAC6 ^cells were generated by treating parental SH-SY5Y cells with an HDAC6-specific shRNA lentiviral clone (clone ID TRCN0000314976, Sigma), which targets the HDAC6 mRNA in the 3'UTR and therefore allows efficient re-transfection with cDNA. 12,500 cells were treated with 1.5 or 0.22 × 10^5 ^transforming units (TU) for 48 h. Selection was performed by adding puromycin (Invivogen) to the culture medium (1 μg/ml).

### Western blot analysis

Cells were collected and lysed in lysis buffer (50 mM Tris (pH 7.4), 50 mM NaCl, 1% NP-40, 0.1% deoxycholate, and 0.1% SDS, 1× Complete proteinase inhibitor (Roche)). Protein concentration was determined by use of bicinchoninic acid (Pierce Biotechnology). Protein was subjected to SDS-PAGE using 4-12% Bis-Tris NuPAGE gradient gels (Invitrogen) and transferred onto nitrocellulose. Membranes were incubated with rabbit anti-TDP-43 (1:2,000, ProteinTech Group), rabbit anti-HDAC6 (1:2,000, Santa Cruz, H-300) or a mouse monoclonal antibody against glyceraldehyde-3-phosphate dehydrogenase (GAPDH) (1:35,000; Biodesign International) overnight followed by horseradish peroxidase-conjugated secondary antibodies (1:15,000; Jackson ImmunoResearch Laboratories). Bands were visualized with ImmobilonWestern Chemiluminescent HRP Substrate (Millipore) on Hyperfilm ECL high performance chemiluminescence (GE Healthcare).

### Neurite outgrowth measurements

*In vitro *differentiation of parental SH-SY5Y cell clones was performed on basis of a previous report with modifications [33]. In brief, 25,000 cells/ml were plated onto poly-D-lysine (Sigma) and collagen (Cohesion) coated cover slips. After overnight incubation, cells were treated with 40 μM RA (Sigma) for 6d. Cells were washed and incubated in serum-free medium containing 50 ng/ml BDNF (Bachem) for another 5d. Cells were fixed, stained and analyzed by microscopy.

For immunostaining, cells were fixed with 4% paraformaldehyde for 20 min at room temperature followed by permeabilization with 1% Triton X-100 in phosphate-buffered saline (PBS) for 30 min. Cells were blocked in 10% normal goat serum and incubated with anti-Flag (1:500, Sigma, M2, affinity purified), anti-Myc (1:500, Roche) or anti-HDAC6 (1:500, Santa Cruz) and/or with Alexa568-coupled phalloidin (Molecular Probes) for 1 h at room temperature followed by incubation with secondary antibody anti-mouse AlexaFluor488 or anti-mouse AlexaFluor647 (both Molecular Probes) for 1 h at room temperature in 1% bovine serum albumin (BSA) in PBS. Cells were washed in PBS and nuclei counterstained with Hoechst 33342 (1:5,000; Sigma) before mounting the cover slips onto slides using fluorescence mounting medium (Dako). Confocal fluorescent images were taken with an AxioImager microscope equipped with an ApoTome Imaging System (Zeiss).

Neurites were quantified using Neurolucida software (Version 8, MBF Bioscience). After manual tracing quantified neurite parameters include total length of neurites (μm), number of neurites and nodes per cell. Mean neurite length was calculated as ratio of total neurite length and number of neurites. Quantified were at least 50 cells per experiment in at least three independent experiments. Statistical analysis was performed with two-sided, paired student's t-test.

### Rescue experiments

Cells were transiently transfected 24 h after RA addition with Myc-HDAC6 or Flag-TDP-43 wt using Lipofectamine 2000 (Invitrogen). Constructs pCMV Myc-HDAC6 and pcDNA3.1(-) Flag-TDP-43 have been described previously [11]. Cells were incubated for additional 72 h with RA-containing medium before fixation, immunofluorescence staining and microscopic analysis.

## List of abbreviations

ALS: amyotrophic lateral sclerosis; BDNF: brain-derived neurotrophic factor; FTLD: frontotemporal lobar degeneration; GAPDH: glyceraldehyde-3-phosphate dehydrogenase; HDAC: histone deacetylase; PBS: phosphate-buffered saline; RA: retinoic acid; shRNA: small hairpin RNA; TDP-43: trans-activation response element (TAR) DNA binding protein of 43kDa; TU: transforming units

## Competing interests

The authors declare that they have no competing interests.

## Authors' contributions

FCF, CS and SSW performed and analyzed the experiments. FCF and PJK designed the study and wrote the paper. PJK is the principal investigator. All authors read and approved the final manuscript.

## Supplementary Material

Additional file 1 **Rescue of stably silenced sh^TDP-43 ^SH-SY5Y cells**. Parental SH-SY5Y cells (Ctrl) or stably transduced cells with shRNA against TDP-43 (shTDP) were transfected with either Flag-TDP-43 wt, Myc-HDAC6 or control vector. Cells were lysed, electrophoresed and Western blots sequentially probed with antibodies against TDP-43 (top panel) and HDAC6 (middle panel). Anti-GAPDH probing (bottom panel) was used as a loading control. A, shown is a representative Western blot. Densitometric analysis of TDP-43 levels B, or HDAC6 levels C, of three independent experiments is shown. Click here for file
