# Supplementary figures and images for: TDP-43 knockdown impairs neurite outgrowth dependent on its target histone deacetylase 6
Source: Mol Neurodegener. 2011 Aug 30;6:64. doi: 10.1186/1750-1326-6-64 (PMC3170629; doi:10.1186/1750-1326-6-64)

A

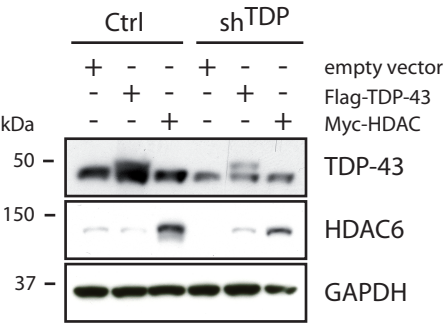

B

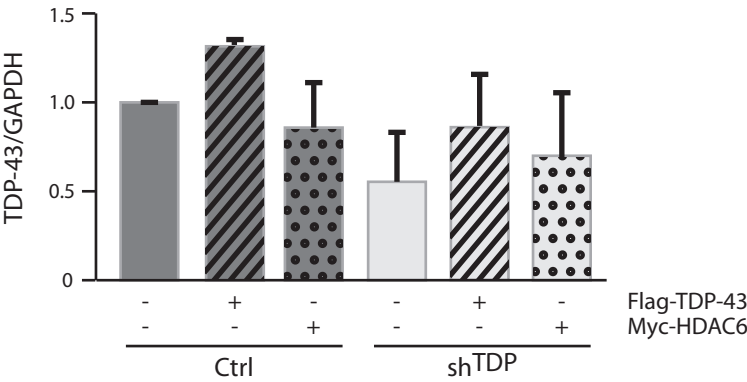

C

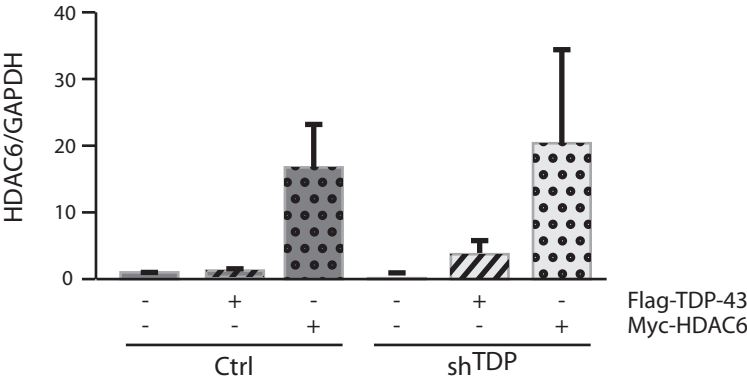

Supplement: Additional file 1 — Rescue of stably silenced shTDP-43 SH-SY5Y cells. Parental SH-SY5Y cells (Ctrl) or stably transduced cells with shRNA against TDP-43 (shTDP) were transfected with either Flag-TDP-43 wt, Myc-HDAC6 or control vector. Cells were lysed, electrophoresed and Western blots sequentially probed with antibodies against TDP-43 (top panel) and HDAC6 (middle panel). Anti-GAPDH probing (bottom panel) was used as a loading control. A, shown is a representative Western blot. Densitometric analysis of TDP-43 levels B, or HDAC6 levels C, of three independent experiments is shown. [file 1750-1326-6-64-S1.PDF]
